# Nicotinamide riboside restores nicotinamide adenine dinucleotide levels and alleviates brain injury by inhibiting oxidative stress and neuroinflammation in a mouse model of intracerebral hemorrhage

**DOI:** 10.1007/s12035-024-04335-w

**Published:** 2024-07-09

**Authors:** Jing She, Hua Zhang, Hui Xu, Yan-Yan Li, Jun-Chao Wu, Rong Han, Fang Lin, Yan Wang, Rui Sheng, Jin-hua Gu, Zheng-Hong Qin

**Affiliations:** 1 Department of Pharmacology and Laboratory of Aging and Nervous Diseases, Jiangsu Key Laboratory of Neuropsychiatric Diseases, College of Pharmaceutical Sciences, Soochow University, Suzhou, 215123 China; 2 Jiangsu Key Laboratory of Neuropsychiatric Diseases, College of Pharmaceutical Sciences, Soochow University, Suzhou, 215123 China; 3 Department of Pharmacy and Nantong Institute of Genetics and Reproductive Medicine, Affiliated Maternity and Child Healthcare Hospital of Nantong University, Nantong, 226018 China; 4 Institute of Health Technology, Global Institute of Software Technology, Qingshan Road, Suzhou Science and Technology Tower, Hi-Tech Area, Suzhou, 215163 China

**Keywords:** Intracerebral hemorrhage, nicotinamide riboside, neuroinflammation, glial activation, oxidative stress

## Abstract

Hemorrhagic stroke is a global health problem owing to its high morbidity and mortality rates. Nicotinamide riboside is an important precursor of nicotinamide adenine dinucleotide characterized by a high bioavailability, safety profile, and robust effects on many cellular signaling processes. This study aimed to investigate the protective effects of nicotinamide riboside against collagenase-induced hemorrhagic stroke and its underlying mechanisms of action. An intracerebral hemorrhage model was constructed by stereotactically injecting collagenase into the right striatum of adult male Institute for Cancer Research mice. After 30 minutes, nicotinamide riboside was administered via the tail vein. The mice were sacrificed at different time points for assessments. Nicotinamide riboside reduced collagenase-induced hemorrhagic area, significantly reduced cerebral water content and histopathological damage, promoted neurological function recovery, and suppressed reactive oxygen species production and neuroinflammation. Nicotinamide riboside exerts neuroprotective effects against collagenase-induced intracerebral hemorrhage by inhibiting neuroinflammation and oxidative stress.

## Introduction

Stroke is an acute cerebrovascular disease that can be ischemic or hemorrhagic. The former is caused by cerebral vascular obstruction, while the latter is a non-traumatic spontaneous intracerebral hemorrhage (ICH) caused by a sudden rupture of the cerebral vasculature [1]. Hemorrhagic stroke, which accounts for approximately 20–25% of all stroke cases, is characterized by high disability and mortality rates. Presently, it is one of the diseases with few effective medical treatments [2]. Therefore, developing new drugs to enhance the removal of blood clots or reduce damage to nerve cells following hemorrhagic stroke is crucial.

ICH can be categorized into primary and secondary ICH, leading to primary and secondary brain injury, respectively [3]. Primary brain injury accounts for 78–88% of all cases and can occur within a few hours of ICH. The leading cause is the rupture of blood vessels. Blood is released into the brain parenchyma and forms a hematoma. The mass effect, compression force, further expansion, and displacement of the formed hematoma compress the peri-hematoma tissues and adjacent neurovascular structures, causing mechanical damage [4]. Therefore, removing hematomas or preventing continuous expansion of hematomas in the initial stages of ICH is an effective treatment method [5].

A series of pathophysiological changes caused by blood clots, known as secondary brain injury [6], plays an important role in the pathophysiological progression of ICH. These pathophysiological changes include but are not limited to oxidative stress injury, neuroinflammation, and blood-brain barrier (BBB) damage, which collectively cause brain cell death, leading to neurological dysfunction.

Following ICH, the whole blood that enters the brain tissue is decomposed by heme oxygenase (HO) into hemoglobin and iron, which are neurotoxic [7]. Hemoglobin-induced iron overload causes the overproduction of reactive oxygen species (ROS), leading to neurotoxicity. ROS is one of the factors that contribute to secondary damage. Neuroinflammation plays a key role in secondary injury following ICH [8, 9]. Neuroinflammation includes cellular and molecular processes, such as glial cell activation, leukocyte infiltration, and proinflammatory factor release, directly causing brain damage. Neuroinflammation is a significant cause of cerebral edema, leading to worsened condition or death in patients with hemorrhagic stroke. The interaction between oxidative stress, neuroinflammation, and other mechanisms causes BBB destruction, loss of neurons, and glial proliferation, accompanied by permanent neurological impairment [10, 11].

Coenzymes I nicotinamide adenine dinucleotide (NAD^+^/NADH) and II nicotinamide adenine dinucleotide phosphate (NADP^+^/NADPH) are known for their essential biological functions. As a hydrogen transmitter, NADPH significantly influences the maintenance of cellular redox homeostasis. NADPH also participates in biological reactions, such as fatty acid synthesis [12]. Our previous studies showed that NADPH significantly influences ischemic stroke by reducing oxidative stress and promoting energy metabolism [13]. NAD^+^ is an important coenzyme involved in energy metabolism in humans. As a hydrogen transmitter in mitochondrial energy synthesis, NAD^+^ maintains redox homeostasis in the body [14]. Previous studies have confirmed that NAD^+^ protects against ischemic stroke by reducing oxidative stress and inhibiting neuroinflammation [15, 16].

NAD^+^ plays an important role in the biosynthesis of NADP^+^, NADH, and NADPH. NAD^+^ level gradually decreases with aging [17]; therefore, it is necessary to supplement the production of NAD^+^. However, exogenous NAD^+^ has low bioavailability owing to severe first-pass effects and polarity; therefore, researchers have turned their attention to the precursors of NAD^+^.

Nicotinamide riboside (NR) is a form of vitamin B3 widely found in meat, eggs, dairy products, and other foods and a precursor to nicotinamide mononucleotide (NMN) in the NAD^+^ salvage synthesis pathway. NR is converted to NMN by nucleoside phosphorylase (NP) or nicotinamide ribose kinase (NRK) in two or one step, respectively. Nicotinamide mononucleotide adenylyltransferase (NMNAT) and NAD^+^ synthetase (NAD_S_) convert NMN to NAD^+^, which can be further transformed into NADPH. Compared to other precursors, NR has a higher bioavailability and safety profile, with the ability to increase NAD^+^ levels; therefore, it has gradually become a candidate precursor [18, 19].

Several studies have confirmed that exogenous NR can increase NAD^+^ levels in the body and protect against various diseases. Han et al. reported that NR protected against aging-induced non-alcoholic fatty liver disease-like liver dysfunction by reducing inflammatory infiltration [20]. Lee et al. reported that NR in AML12 hepatocytes regulates neuroinflammation and mitochondrial function [21]. Vaur et al. reported that NR protects against axonal degeneration induced by excitatory toxicity [22]. Hong et al. reported that NR protects against liver injury during sepsis by inhibiting oxidative stress [23]. Li et al. found that NR can improve mitochondrial function by activating the AMPK pathway in skeletal muscle, which can help improve insulin resistance [24]. Moreover, NR reportedly plays a protective role against sepsis and other diseases by inhibiting neuroinflammation and maintaining oxidative stress homeostasis. We hypothesized that NR may protect against hemorrhagic stroke, possibly by inhibiting neuroinflammation and oxidative stress.

In this study, we conducted a direct investigation to evaluate if replenishing NAD^+^ using NR might increase the NAD^+^ levels in the brain and protect against collagenase-induced hemorrhagic stroke, and if so, to further explore the potential molecular mechanisms of NR for the treatment of ICH.

## Materials and methods

### Animals

Specific pathogen-free adult male Institute of Cancer Research (ICR) mice (25–28 g) were obtained from Zhao Yan (Suzhou) New Drug Research Company (experimental animal license number SCXK (Su) 2019-0006). All animal experiments were performed in accordance with the institutional guidelines for animal use and care, and all experimental procedures were approved by the Ethics Committee of Soochow University. Animal feeding conditions were as follows: temperature, 22°C; humidity, 50–60%; good ventilation environment; and artificial day and night (12 h/12 h). All the animals had free access to food and water during the experiment.

### ICH mice model

The specific preparation procedures were as follows: the mice were anesthetized with 2.5% isoflurane and maintained with 1.5% isoflurane during the entire surgical procedure. Anesthetized mice were fixed in a stereotaxic apparatus. An incision was made on the heads of the mice to expose their skulls. A microsyringe (World Precision Instruments, LLC, Sarasota, FL, USA) was used to slowly inject 0.5 μL collagenase VII (0.06 U/μL, Sigma-Aldrich, USA) into the right striatum (position: the anterior fontanelle is zero, x = -1.8 mm, y = 0.8 mm, z = -3.5 mm) at a rate of 0.125 μL/min. The needle was left in place for 5 min after the injection. The mice were put on an electric blanket (maintained at 37°C) until they woke up and were thereafter put back into the cage. Food and water were provided ad libitum. Based on the experimental design, the animals were sacrificed at different time points following ICH.

### Experimental design and groups

Healthy mice (25–28 g) were randomly assigned to different groups. The model groups comprised ICH (1.5 h), ICH (3 h), ICH (6 h), ICH (12 h), ICH (24 h), ICH (72 h), and sham groups while the treatment groups comprised ICH + NR (75 mg/kg), ICH + NR (150 mg/kg), ICH + NR (300 mg/kg), ICH + NR (600 mg/kg), and ICH + NR (1200 mg/kg) groups. NR was administered via the tail vein 30 min following ICH once in 24 h. Brain tissues around the hematoma were collected at corresponding time points for follow-up experiments.

### Measurement of cerebral water content

The animals were sacrificed 24 and 72 h after receiving collagenase injection. The brainstem, cerebellum, and olfactory bulb were excised. The brain sections were placed in a drying oven maintained at 37°C for >48 h to measure water content [(wet weight-dry weight)/wet weight] × 100 %.

### Measurement of cerebral hematoma area

The animals were sacrificed at corresponding time points after receiving collagenase injection. Subsequently, the cerebellum and olfactory bulb were removed, and only the brain was retained. The brains were cut vertically into five uniform coronal sections. The bleeding area was red, whereas the non-bleeding area was white. The brains were scanned using a multifunctional scanner. The percentage of the tissue in the bleeding area to the entire brain tissue area was used as an index to measure the cerebral hematoma area.

### Western blot analysis

After the animals were sacrificed, the brain tissues around the hematoma were collected and added to 200 μL cell lysate. Next, the brain tissues were lysed on ice for >40 min using an ultrasonic cell crushing apparatus, and the supernatant was extracted after centrifugation. A bicinchoninic acid kit was used to determine the protein concentration, and all samples were adjusted to the same concentration. The samples were mixed and heated in a boiling water bath for 5 min to denature the proteins.

### Measurement of NAD^+^ levels

The mice in the control, ICH, and ICH + NR groups were assessed 2 h, 4 h, and 6 h after receiving NR, respectively (2.5 h, 4.5 h, and 6.5 h, respectively after collagenase injection). The mice were sacrificed; tissues at the right side of the brain around the hematoma were collected and washed using cold phosphate buffer saline (PBS). Thereafter, 100 μL of NAD^+^ extraction buffer was added to every 20 mg of tissue for NAD^+^ level determination. The extract was heated at 60°C for 5 min, followed by the addition of 20 μL assay buffer and 100 μL of the opposite extraction buffer to neutralize the extracts. The samples were then vortexed and centrifuged at 14000 rpm for 5 min. The supernatant was used for the NAD^+^ content assay according to the NAD^+^/NADH detection kit manufacturer’s instructions (Beyotime, Nantong, China).

### Measurement of malondialdehyde (MDA), reduced glutathione (GSH)/ oxidized glutathione (GSSG), superoxide dismutase (SOD), adenosine triphosphate (ATP), and hydrogen peroxide (H_2_O_2)_ levels

The mice were sacrificed 24 h after collagenase injection, and the right side of the brain tissue around the hematoma was collected to measure MDA, GSH/GSSG, SOD, ATP, and H_2_O_2_ levels. MDA, GSH/GSSG, SOD, ATP, and H_2_O_2_ levels were determined using MDA, GSH/GSSG, SOD, ATP, and H_2_O_2_ assay kits (Beyotime, Nantong, China) according to the manufacturer's instructions.

### Real-time quantitative polymerase chain reaction (PCR)

After the animals were sacrificed, brain tissues around the hematoma were collected. Total RNA was extracted from the peri-hematoma tissues using RNAiso Plus reagent (Takara, Tokyo, Japan). The total RNA (500 ng) was reverse transcribed into cDNA using PrimeScript™ RT Master Mix (Takara, Tokyo, Japan). Real-time quantitative PCR was performed using TB Green® Fast qPCR Mix (Takara, Tokyo, Japan) and ABI-7500 quantitative PCR system (Applied Biosystems, Warrington, UK). The primer sequences used for RT-PCR analysis of specific genes are listed in Table 1.

**Table 1:** A list of primer sequences for RT-PCR analysis

Gene	Forward	Reverse
m-β-actin	TCAGCAAGCAGGAGTACGATG	GTGTAAAACGCAGCTCAGTAACA
m- TNF-α	CATCTTCTCAAAATTCGAGTGACAA	TGGGAGTAGACAAGGTACAACCC
m- IL-1β	CCTATGTCTTGCCCGTGGAG	CACACACTAGCAGGTCGTCA
m-NLRP3	CGGTGACCTTGTGTGTGCTT	TCATGTCCTGAGCCATGGAAG
m-ASC	GACAGTACCAGGCAGTTCGT	AGTAGGGCTGTGTTTGCCTC
m-Caspase-1	GAACAAAGAAGGTGGCGCAT	AGACGTGTACGAGTGGGTGT

### Immunohistochemistry

The mice were anesthetized and perfused with cold PBS and 4% paraformaldehyde 24 h after receiving collagenase injection. The mice were sacrificed, and sections of their brain were collected. The sections were immersed in 4% paraformaldehyde at 4°C for 2 days and dehydrated with 20–30% gradient sucrose solution. The dehydrated brains were frozen at -80 °C, and tissues around the hematoma were cut into slices of 20 μm in thickness using a cryostat microtome (CM1950 Freezing Microtome, Leica, Wetzlar, Germany). Thereafter, the sections were blocked with 5% horse serum (containing 0.5% Triton X-100) and incubated with primary antibodies for 48 h at 4°C. The sections were incubated with secondary antibodies for 2 h at room temperature (22–24°C), and the nuclei were stained with 4, 6-diamino-2-phenylindole for 15 min at room temperature. Finally, the sections were photographed, and ImageJ software was used to analyze fluorescence intensity.

### Hematoxylin and Eosin (HE) staining

The mice were treated with heart perfusion 24 h after receiving collagenase injection, immersed in a 50 mL tube containing 4% paraformaldehyde, and fixed in a refrigerator at 4°C. After fixation, the mice were embedded in paraffin, and the brain section thickness was determined to be 4 μm. After sectioning and dewaxing, HE staining was performed. Thereafter, the sections were dehydrated and sealed. Finally, images were collected and analyzed under a microscope (the colors of the nucleus and cytoplasm were blue and red, respectively).

### Neurobehavioral tests

The improved 24-point neurological dysfunction score (NDS) was used to determine the degree of neurological impairment in each mouse 24 and 72 h after administering collagenase. The NDS consists of six parts, each of which scores 0–4 points. A score of 0 and 4 indicated no impairment and the most severe neurological impairment, respectively.

### Beam balance experiment

The mice were trained on the day before the formal balance-beam experiment. The mice were placed on one end of the balance beam and instructed to crawl independently from one end to the other. The mice were trained several times, with a 2-hour interval between each training session, and were given sufficient rest until they learned to crawl across the balance beam independently.

The mice were placed on one end of the balance beam, and the time needed to crawl from one end of the balance beam to the other was recorded (short stays on the balance beam were not recorded). The balance beam was wiped with a rag and disinfected with 75% alcohol to remove odor and urine after each crawling. Each mouse was tested thrice, and the average value was calculated.

### Successful steps experiment

The mice were made to touch the ground with their whiskers 10 times. The number of times the mice placed their forelimbs on the table and edge of the table in response to whisker stimulation was determined.

### Right turn experiment

The mice were placed in a manually created 30 ° corner and were allowed to turn left or right. Each mouse was tested 10 times, and the number of turns from the right side was recorded to determine the percentage of turns from the right side.

### Statistical analysis

The results of all experiments are presented as mean ± standard error of the mean (SEM). One-way analysis of variance (ANOVA) followed by Student–Newman–Keuls post-hoc test were used for multiple group comparisons. Two-way ANOVA followed by Bonferroni's post-hoc test were used to analyze cerebral hematoma data. The significance of the differences between the groups was determined using GraphPad Prism 8.0 software (GraphPad Software, San Diego, USA). Differences were considered statistically significant at *P* < 0.05.

## Results

### NR reduced the hemorrhagic area and cerebral water content following ICH

Collagenase-induced ICH and cerebral edema

**Fig. 1:**
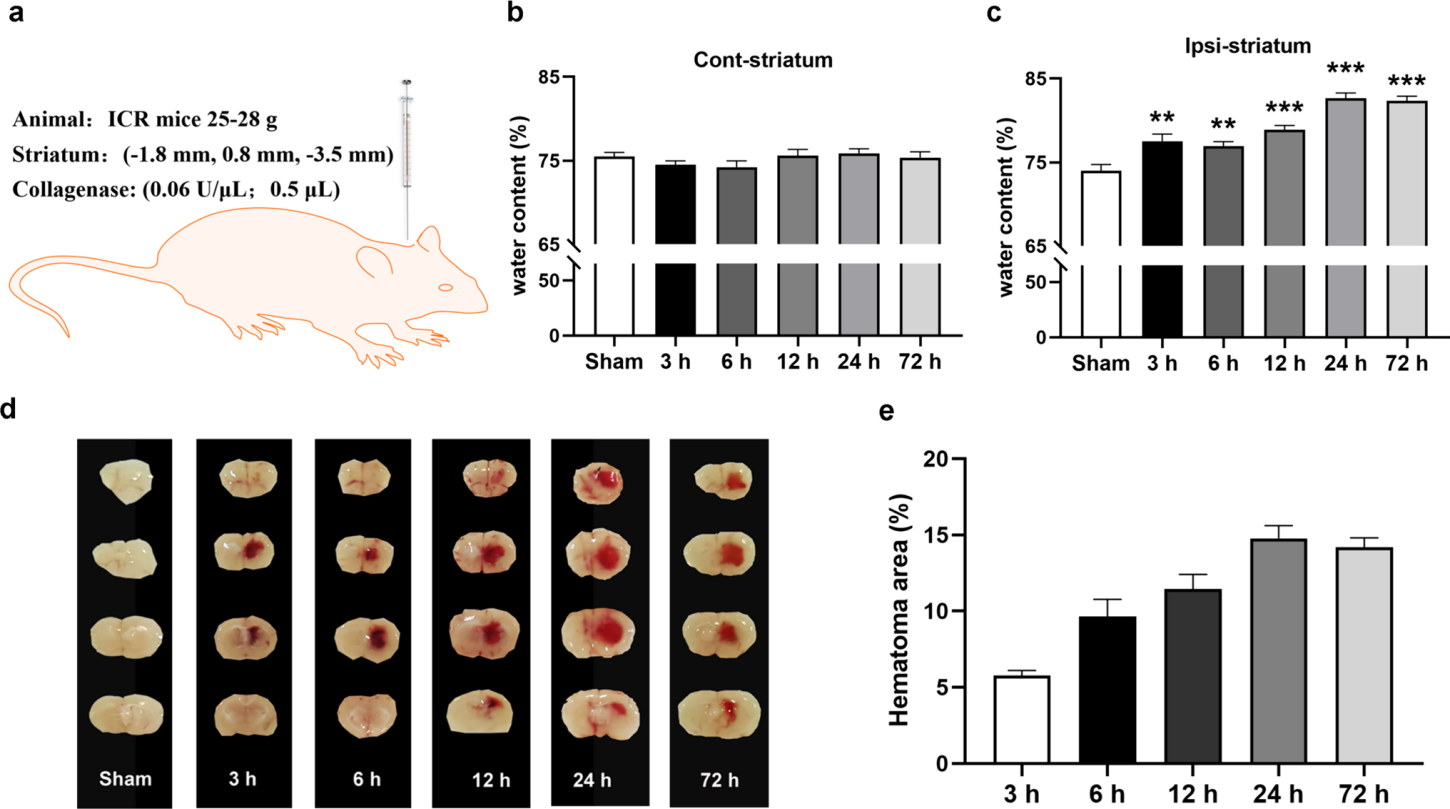
Collagenase-induced ICH and cerebral edema

A mice model of cerebral hemorrhage was constructed by stereotactically injecting collagenase (0.03 U) into the striatum of adult male ICR mice (25–28 g) (Fig. 1a). The mice were sacrificed at 3, 6, 12, 24, and 72 h to observe brain hemorrhage. The brain water content and hematoma areas were examined. The results showed that the brain water content of the mice injected with collagenase significantly increased after 3, 6, 12, 24, and 72 h compared to that in the sham group (Fig. 1b and c). A hematoma was observed in the brain 3 h after collagenase was administered, and the size of the hematoma at 24 h was the largest (Fig. 1d and e). These results demonstrate that the ICH model was successfully constructed. The brain water content and hematoma area results indicated that the maximum damage caused by collagenase occurred after 24 h or 72 h.

(a) Experimental timeline and flow diagram. The mice were sacrificed at 3, 6, 12, 24, and 72 h after collagenase was administered. (b and c) Time course of collagenase-induced cerebral edema. (d and e) Time course of collagenase-induced ICH. Data are expressed as mean ± SEM (n = 6, ^**^ *P* < 0.01, ^***^ *P* < 0.001 vs. Sham, one-way ANOVA followed by Student–Newman–Keuls post-hoc test).

**Fig. 2:**
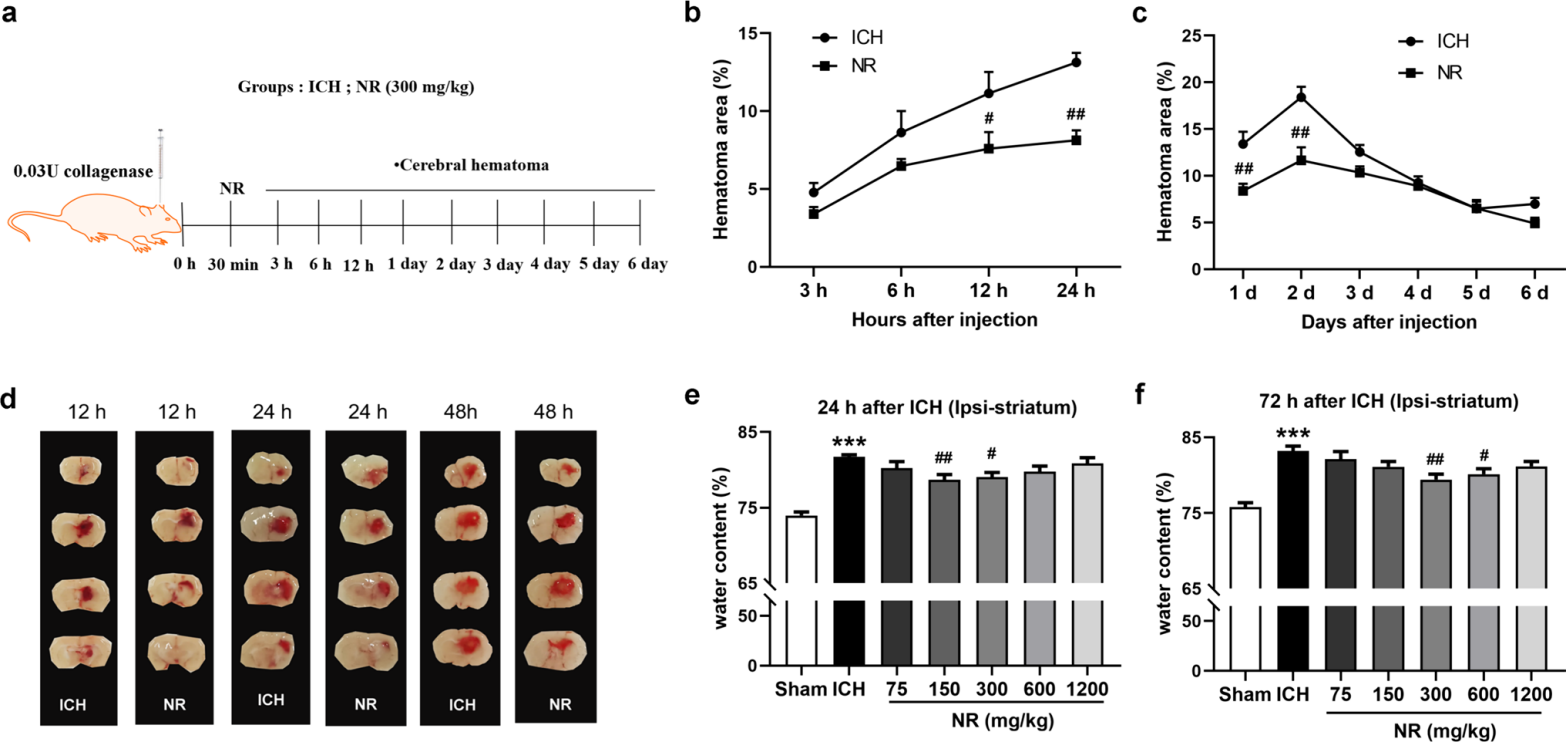
NR reduced the hemorrhagic area and cerebral water content following ICH

To evaluate the effect of NR on primary and secondary brain injury following ICH, the mice were sacrificed at 3, 6, 12, and 24 h and on days 2, 3, 4, 5, and 6 following ICH (Fig. 2a). The cerebral hematoma area was measured, and the results showed that NR reduced the hemorrhagic area and enhanced hematoma clearance (Fig. 2b–d). The cerebral water content of the ipsilateral striatum of the mice in ICH groups was significantly higher than that in mice in the sham group. The cerebral water content of the mice ipsilateral striatum was reduced by 150 mg/kg and 300 mg/kg of NR after 24 h (Fig. 2e) and by 300 mg/kg and 600 mg/kg of NR after 72 h (Fig. 2f).

(a) Experimental design timeline. For 6 consecutive days, mice were administered 300 mg/kg of NR once daily intravenously 30 min after receiving collagenase injection. The mice were sacrificed after 3 h, 6 h, 12 h, 24 h, and on days 2, 3, 4, 5, and 6. (b and c) Time course of the effects of NR on cerebral hematoma following ICH. (d) Representative images of the effect of NR on cerebral hematoma 12, 24, and 48 h following ICH. (e) Effect of NR on cerebral water content 24 h following ICH. (f) Effect of NR on cerebral water content 72 h following ICH. Data are expressed as mean ± SEM (n = 5, ^#^ *P* < 0.05, ^##^ *P* < 0.01 vs. ICH, one-way ANOVA followed by Student–Newman–Keuls post-hoc test, and two-way ANOVA followed by Bonferroni's post-hoc test).

### Exogenous NR restored NAD^+^ level following ICH

**Fig. 3:**
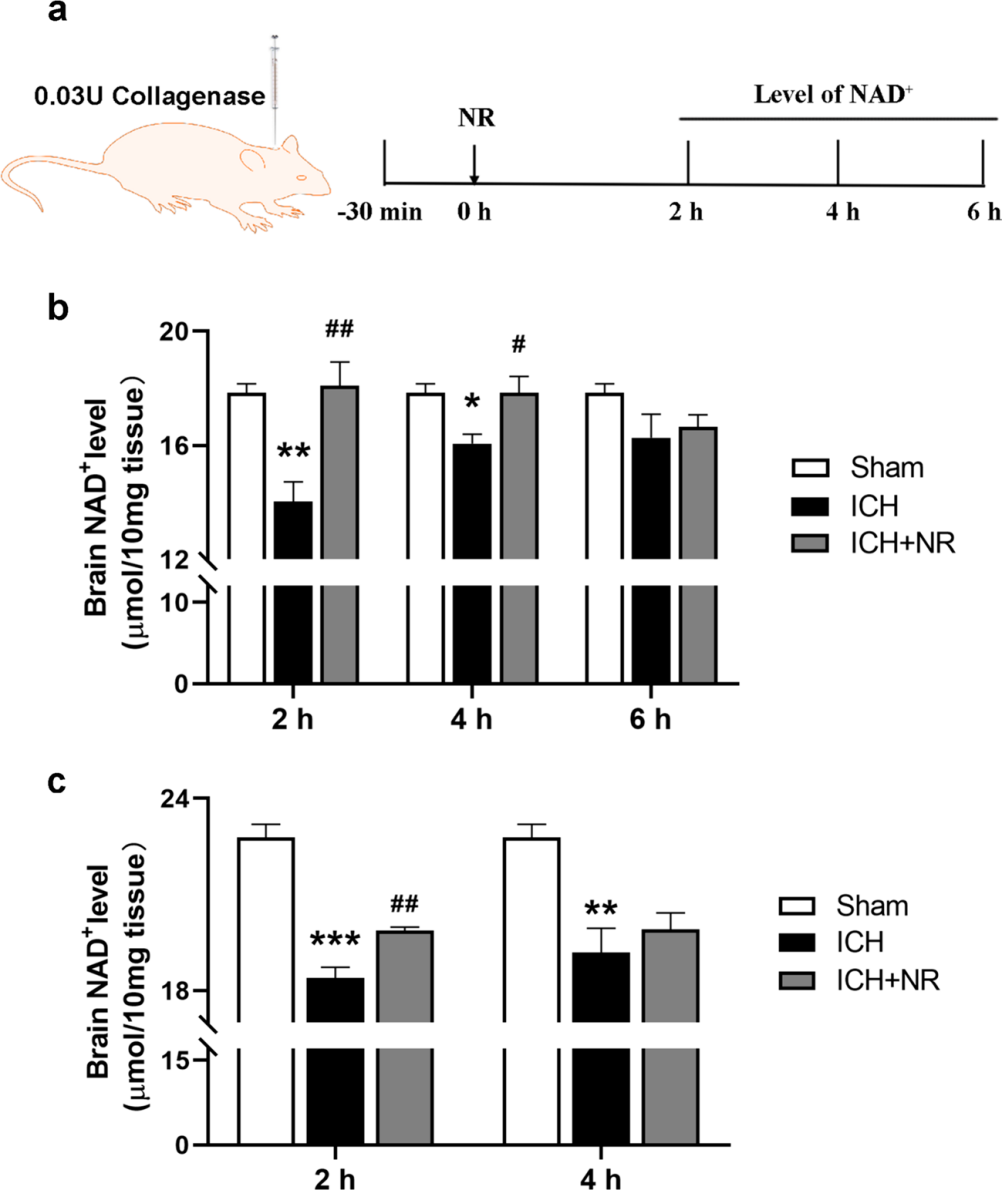
Exogenous NR restored NAD^+^ levels following ICH

We examined whether exogenous NR could increase NAD^+^ levels in the brain. The mice were randomly assigned into seven groups: control, ICH (2 h), ICH + NR (2 h), ICH (4 h), ICH + NR (4 h), ICH (6 h), and ICH + NR (6 h) groups. Peri-hematoma tissues were collected to determine NAD^+^ level (Fig. 3a). The results showed a significant decrease and increase in NAD^+^ levels in the peri-hematoma tissues of the mice in the ICH and ICH + NR groups, respectively, after 2 h and 4 h (Fig. 3b), suggesting that exogenous NR can restore NAD^+^ level in the peri-hematoma tissues following ICH.

To exclude the influence of blood NAD^+^ level on NAD^+^ levels in the brain tissue, the mice were perfused in the sham, ICH (2 h), ICH + NR (2 h), ICH (4 h), and ICH + NR (4 h) groups before undergoing brain dissection. The results showed a significant decrease and increase in NAD^+^ levels in the peri-hematoma tissues of the mice in the ICH and ICH + NR groups, respectively, after 2 and 4 h (Fig. 3c), further proving that exogenous NR can restore NAD^+^ levels in the peri-hematoma tissues following ICH.

(a) Experimental design timeline. The mice in the model groups were administered 300 mg/kg of NR intravenously 30 min following ICH, whereas those in the control groups were administered normal saline. The mice were sacrificed 2, 4, and 6 h after NR was administered. (b) Time course of changes in NAD^+^ concentration in the brain. (c) Time course of changes in NAD^+^ concentration in the brain after myocardial perfusion. Data are expressed as mean ± SEM (n = 5, ^*^ *P* < 0.05, ^**^ *P* < 0.01, ^***^ *P* < 0.001 vs. Control; ^#^ *P* < 0.05, ^##^ *P* < 0.01 vs ICH, one-way ANOVA followed by Student-Newman–Keuls post-hoc test).

### NR improved behavioral deficits 24 and 72 h following ICH

**Fig. 4:**
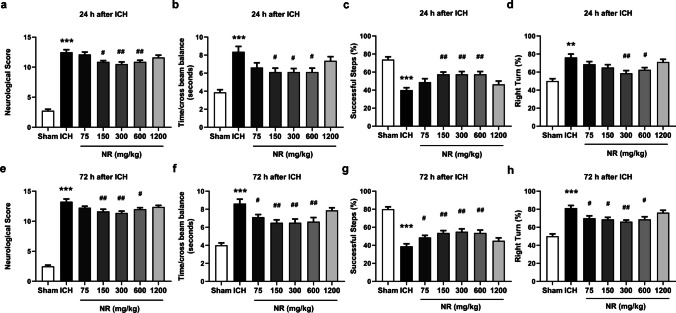
NR ameliorated behavioral deficits 24 and 72 h following ICH

Administered to explore whether NR has a protective effect on the neurological function of mice with hemorrhagic stroke. We used a double-anonymized method to evaluate neurological deficits, motor balance, motor ability, and limb muscle tension. The results showed that among mice in the ICH group, defective nerve symptoms were significantly worse (Fig. 4a), time spent crawling from one end of the beam to the other was significantly increased (Fig. 4b), frequency with which the forelimb after the antennae was placed on the table was significantly reduced (Fig. 4c), and asymmetrical sensorimotor deficiency worsened (Fig. 4d) after 24 h. To a certain degree, 150, 300, and 600 mg/kg of NR ameliorated neurological deficits, reduced the time it took to crawl from one end of the balance beam to the other, increased the number of times the forelimb was placed on the table after the tentacles, thus ameliorating neurological deficit and improving motor balance ability and limb muscle tension. Neurological function was assessed after 72 h, and similar results were obtained (Fig. 4e–h).

After 30 min of receiving collagenase injection to induce ICH, the mice were administered 75, 150, 300, 600, or 1200 mg/kg NR intravenously for 3 consecutive days, and were sacrificed after 24 and 72 h. Effects of NR on (a) neurological deficit, (b) beam balance movement, (c) forelimb placement, and (d) corner test results 24 h following ICH. Effects of NR on (e) neurological deficit, (F) beam balance movement, (g) forelimb placement, and (h) corner test results 72 h following ICH. Data are expressed as mean ± SEM (n = 8, ^***^ *P* < 0.001 vs. Sham; ^#^ *P* < 0.05, ^##^ *P* < 0.01 vs. ICH, one-way ANOVA followed by Student–Newman–Keuls post-hoc test).

### NR alleviated histological damages 24 h following ICH

**Fig. 5:**
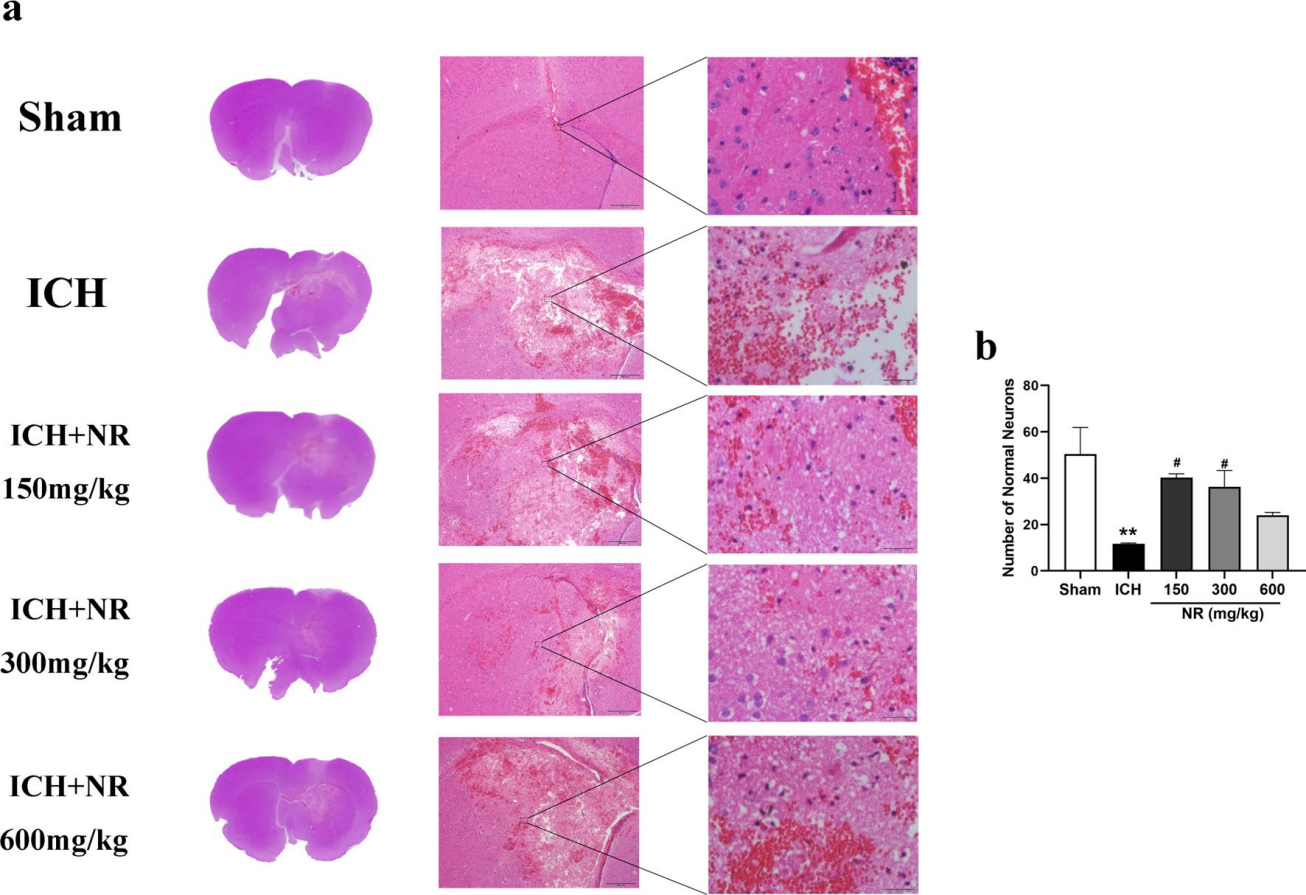
NR alleviated histological damages 24 h following ICH

We investigated the effects of NR on brain histopathology following ICH. The results showed that the mice in the ICH group had a significant hemorrhage on the right side of the brain, loose tissue around the hematoma, and a significantly reduced number of normal neurons. NR (150, 300, and 600 mg/kg) improved the morphology of the tissue around the hematoma, and significantly increased the number of normal neurons in the peri-hematoma tissues (Fig. 5).

Mice were administered 150, 300, or 600 mg/kg of NR intravenously 30 min following ICH. The effects of NR on brain histopathology were observed 24 h following ICH. (a) Histopathological changes of brain were evaluated by HE staining. Scale bar = 500 μm, 50 μm. (b) number of normal neurons. Data are expressed as mean ± SEM (n = 6, ^***^ *P* < 0.001 vs. Sham; ^#^ *P* < 0.05, ^##^ *P* < 0.01 vs. ICH, one-way ANOVA followed by Student–Newman–Keuls post-hoc test).

### NR suppressed the components of the NLRP3 inflammasome pathway following ICH

**Fig. 6:**
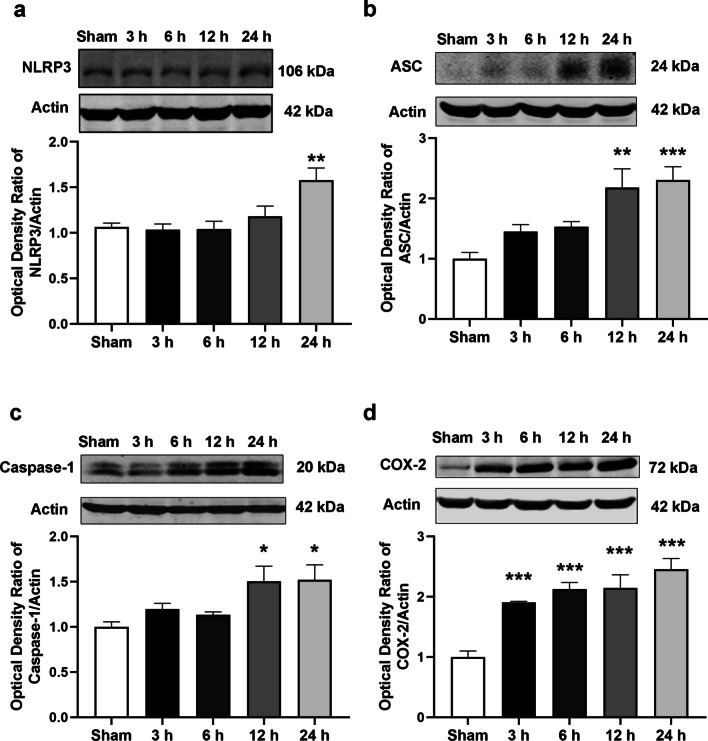
Collagenase-induced neuroinflammation

To determine whether collagenase could induce neuroinflammation, the mice were sacrificed at 3, 6, 12, and 24 h following ICH. Western blot analysis showed that NLRP3, ASC, and Caspase-1 levels significantly increased after 12 and 24 h (Fig. 6a–c). The results also showed that cyclooxygenase (COX)-2 levels increased dramatically with time (Fig. 6d), suggesting that neuroinflammation was activated 12 and 24 h following ICH.

Changes in the levels of (a) NLRP3, (b) ASC, (c) Caspase-1, and (d) COX-2 at different time points following ICH. Data are expressed as mean ± SEM (n = 5, ^*^ *P* < 0.01, ^**^ *P* < 0.01, ^***^ *P* < 0.001 vs. Sham, one-way ANOVA followed by Student-Newman–Keuls post-hoc test).

**Fig. 7:**
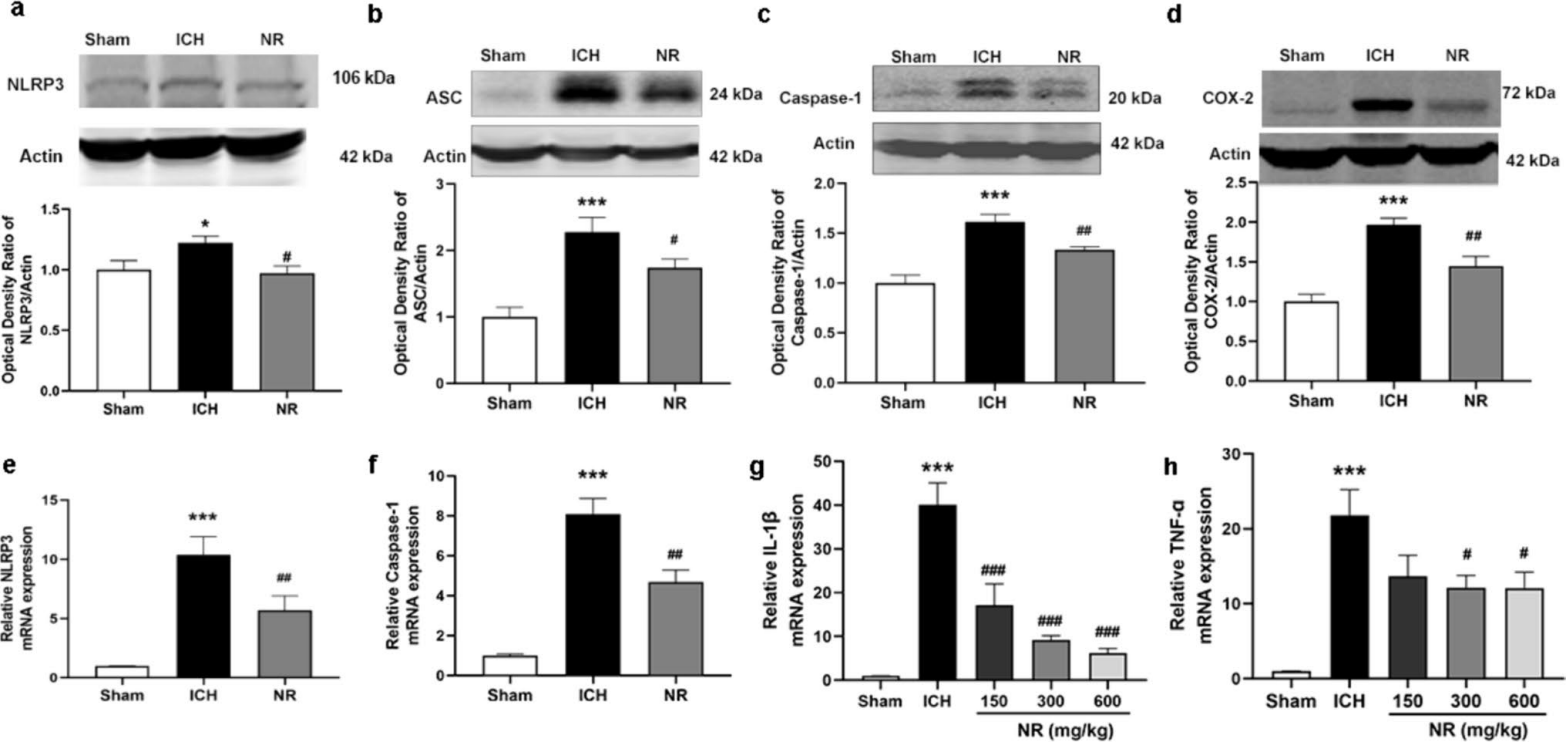
NR suppressed the components of NLRP3 inflammasome pathway following ICH

NLRP3, ASC, and Caspase-1 levels in the peri-hematoma tissues were measured to assess whether NR plays a protective role by inhibiting inflammasome activation. Western blot results showed significantly increased protein levels of NLRP3, ASC, and Caspase-1 following ICH (Fig. 7a–c) and mRNA levels of NLRP3 and Caspase-1 (Fig. 7e and f). Protein and mRNA levels of NLRP3, Caspase-1, and ASC were significantly reduced by 300 mg/kg of NR.

The levels of COX-2, tissue-necrosis factor (TNF-α), and interleukin (IL)-1β in the peri-hematoma tissues were measured. PCR and Western blot results showed significantly increased protein levels of COX-2 and mRNA levels of IL-1β and TNF-α among mice in the ICH group compared with those in the sham group (Fig. 7d, g, and h). mRNA levels of IL-1β and TNF-α and protein levels of COX-2 were significantly reduced by 300 mg/kg of NR, suggesting that NR plays a protective role against neuroinflammation.

Effects of NR on (a) NLRP3, (b) ASC, and (c) Caspase-1 protein levels in the peri-hematoma tissue 24 h following ICH. (d) Effect of NR on COX-2 protein levels in the peri-hematoma tissues 24 h following ICH. Effects of NR on (e) NLRP3 and (f) Caspase-1 mRNA levels in the peri-hematoma tissue 24 h following ICH. Effects of NR on (g) IL-1β and (h) TNF-α mRNA levels in the peri-hematoma tissues 24 h following ICH. Data are expressed as mean ± SEM (n = 6, ^**^ *P* < 0.01, ^***^ *P* < 0.001 vs. Sham; ^#^ *P* < 0.05, ^##^ *P* < 0.01 vs. ICH, one-way ANOVA followed by Student–Newman–Keuls post-hoc test).

### NR inhibited astrocyte and microglia activation in the peri-hematoma tissues

**Fig. 8:**
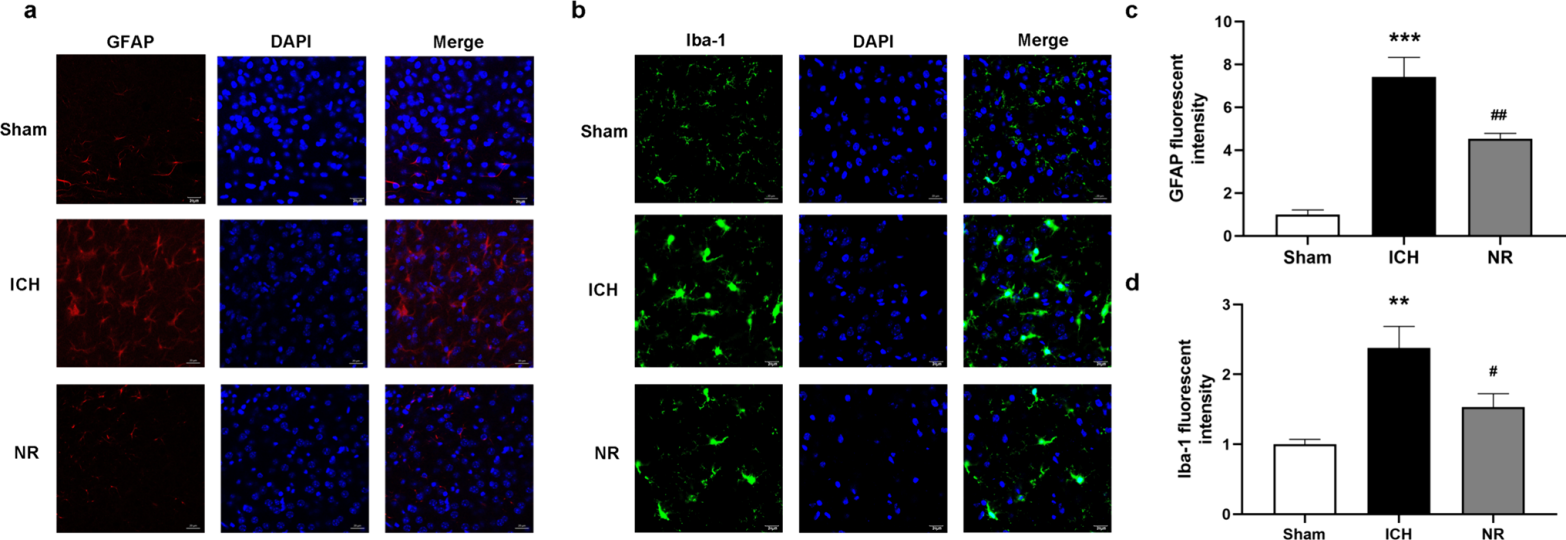
NR inhibited astrocyte and microglia activation in the peri-hematoma tissues

The effect of NR on glial cells was evaluated by detecting the activation of glial fibrillary acidic protein (GFAP) and ionized calcium-binding adapter molecule (Iba-1) in the peri-hematoma tissues. The number of GFAP-positive cells increased, the cell bodies became smaller, and the branches became longer, suggesting the activation of astrocytes (Fig. 8a). The results also showed that the number of Iba-1 positive cells increased, cell bodies became larger, and cell branches became shorter, suggesting significant microglia activation (Fig. 8b). Morphological alterations in astrocytes and microglia were significantly inhibited by 300 mg/kg of NR (Fig. 8c and d).

Mice received 300 mg/kg of NR intravenously 30 min following ICH. (a, c) Effect of NR on astrocyte activation in the peri-hematoma tissues 24 h following ICH. (b, d) Effect of NR on microglia activation in the peri-hematoma tissues 24 h following ICH. Scale bar = 20 μm. Data are expressed as mean ± SEM (n = 6, ^**^ *P* < 0.01, ^***^ *P* < 0.001 vs. Sham; ^#^ *P* < 0.05, ^##^ *P* < 0.01 vs. ICH, one-way ANOVA followed by Student–Newman–Keuls post-hoc test).

### NR treatment mitigated oxidative stress through the Nrf2/HO-1 pathway following ICH

**Fig. 9:**
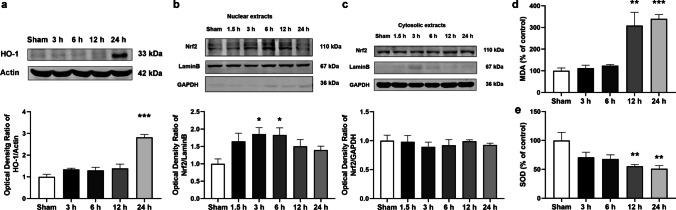
Collagenase-induced oxidative stress following ICH

During ICH, oxidative stress contributes significantly to the aggravation of neurological damage. The mice were sacrificed 3, 6, 12, and 24 h following ICH. Western blot was performed to detect HO-1 protein levels. The results showed that HO-1 protein levels significantly increased 24 h following ICH (Fig. 9a). Nuclear respiratory factor -2 (Nrf2) is an upstream regulator of heme oxygenase -1 (HO-1) and a key regulatory factor in the oxidative stress process. Mice were sacrificed at 1.5, 3, 6, 12, and 24 h following ICH to detect Nrf2 protein levels. The results showed that Nrf2 protein levels were significantly increased in the nucleus after 3 and 6 h (Fig. 9b and c). MDA content (Fig. 9d) and SOD activity (Fig. 9e) were measured to evaluate the effects of collagenase on oxidative stress. SOD activity significantly decreased after 12 and 24 h, whereas MDA content significantly increased, suggesting that collagenase injection induced oxidative stress.

Protein levels of (a) HO-1 and (b and c) Nrf2 at different time points following ICH. (d) MDA content and (e) SOD activity at different time points following ICH. Data are expressed as mean ± SEM (n = 5, ^*^ *P* < 0.05, ^**^ *P* < 0.01, ^***^ *P* < 0.001 vs Sham, one-way ANOVA followed by Student–Newman–Keuls post hoc test).

**Fig. 10:**
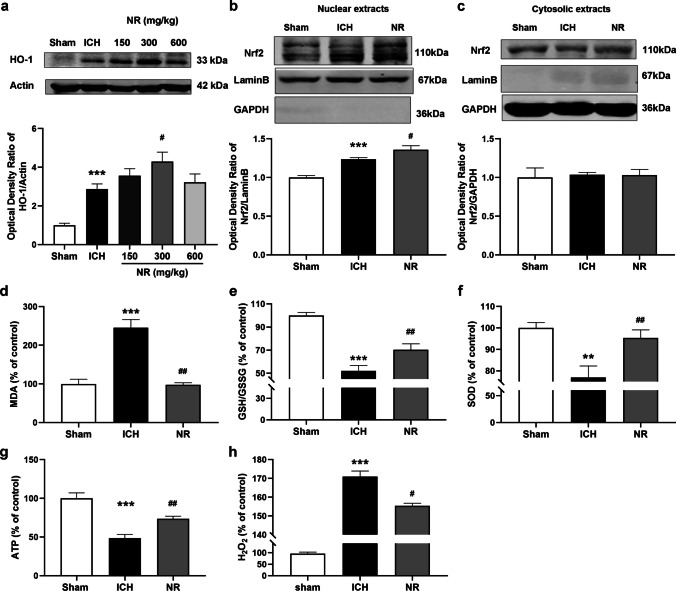
NR mitigated oxidative stress through Nrf2/HO-1 pathway after ICH

To explore whether NR exerts a protective effect by improving anti-oxidative stress, we measured the levels of the antioxidant protein, HO-1 and nuclear transcription of Nrf2 in the peri-hematoma tissues. Western blot analysis showed that HO-1 protein levels and Nrf2 nuclear localization significantly increased in mice in the ICH group, whereas NR further increased HO-1 protein levels (Fig. 10a) and promoted Nrf2 nuclear localization (Fig. 10b).

The levels of MDA, GSH/GSSG, SOD, ATP, and H_2_O_2_ in the peri-hematoma tissues were also determined. Significantly increased MDA and H_2_O_2_ levels (Fig. 10d and h) and decreased GSH/GSSG, SOD, and ATP levels were observed in the ICH group (Fig. 10e-g). However, MDA and H_2_O_2_ levels were significantly reduced, and GSH/GSSG, SOD, and ATP levels were significantly increased by 300 mg/kg of NR. These results further demonstrated that NR plays a protective role by promoting anti-oxidative stress and maintaining energy metabolism.

(a) Effect of NR on HO-1 protein levels in the peri-hematoma tissues 24 h following ICH. (b-c) Effect of NR on Nrf2 protein levels in the nucleus and cytoplasm 24 h following ICH. Mice were administered 300 mg/kg intravenously 30 min following ICH. Effects of NR on MDA level (d), GSH/GSSG ratio (e), SOD activity (f), ATP (g), and H_2_O_2_ levels (h) in the peri-hematoma tissues 24 h following ICH. Data are expressed as mean ± SEM (n = 6, ^***^ *P* < 0.001 vs. Sham; ^#^ *P* < 0.05 vs. ICH, one-way ANOVA followed by Student–Newman–Keuls post hoc test).

## Discussion

This study is the first to test the therapeutic potential of NR against ICH-induced brain injury. In this study, we constructed an ICH mouse model by stereotactically injecting collagenase into mice's ipsilateral striata. We found that collagenase-induced brain hemorrhage and oxidative stress and promoted neuroinflammation (via NLRP3 inflammasome), which aggravated brain damage. On the other hand, NR effectively inhibited brain edema, promoted anti-oxidative stress, and reduced neuroinflammation. Finally, we found that NR treatment upregulated the expression of Nrf2 and HO-1 proteins and promoted nuclear translocation of Nrf2. Based on these results, we propose that NR is an effective therapeutic agent for ICH.

Hemorrhagic stroke accounts for approximately 20–25% of stroke cases. Hemorrhagic stroke is caused by an acute hemorrhage in the brain, leading to a blood clot that causes insufficient blood supply and brain edema. Currently, the treatment methods for hemorrhagic stroke are reducing brain edema and removing blood mass by craniotomy. However, craniotomy has a poor long-term clinical prognosis, affects the recovery of neurological function [25], and has a high postoperative risk. No targeted drug therapy exists for hemorrhagic stroke.

Cerebral edema, a secondary brain injury, has a considerable effect on the physiological and pathological development of ischemic cerebral and secondary brain injuries as it promotes an increase in intracranial pressure and aggravates brain damage [26]. Two mechanisms are involved in the development of cerebral edema. The first is vasogenic brain edema involving a series of physiological and pathological changes caused by ICH, such as hydrostatic pressure, which can lead to the infiltration of serum proteins into the peri-hematoma tissues and activation of the coagulation system to produce thrombin. These changes promote neuroinflammation and destruction of the BBB, increase the levels of inflammatory factors, and increase the exudation of iron and water, eventually leading to vasogenic cerebral edema. The second is cytotoxic cerebral edema induced by hemoglobin cytotoxicity [27].

Studies on ischemic stroke have shown that excessive ROS production not only causes oxidative damage following cerebral ischemia [28] but also activates inflammatory apoptotic pathways, enhancing cerebral ischemic injury [29]. Studies have demonstrated that reducing oxidative stress ameliorates ischemic brain injury [30]. Recently, increasing evidence suggests that oxidative stress and inflammation play critical pathogenic roles in ICH [31].

In 2002, Martinon first proposed the inflammasome as a multiprotein complex composed of NLRP3, ASC, and Caspase-1 [32]. NLRP3 is the most studied inflammasome. NLRP3 activation comprises two steps. Pathogen-associated and damage-associated molecular patterns are first recognized by pattern recognition receptors, which initiate the nuclear translocation of NF-κB and promote the transcription of NLRP3, IL-1β, and IL-18. Subsequently, Caspase-1 is activated and cleaves the IL-1β and IL-18 precursors to produce mature extracellular forms of IL-1β and IL-18. Aberrant NLRP3 activation leads to excessive inflammation and aggravation of neuroinflammation.

NAD^+^ is involved in various biological processes, including cellular activity, energy metabolism, oxidative stress, and aging [33–35]. NAD^+^ has neuroprotective effects that regulate nerve cell survival and improve nerve function [36]. NAD^+^ is synthesized at physiological conditions; however, under pathological conditions, TNF-α and oxidative stress can significantly reduce the levels of NAMPT and NAD^+^ [37]. Therefore, exogenous NAD^+^ has considerable potential in treating various neurodegenerative diseases. NA, NAM, NMN, and NR have been publicized as effective NAD^+^ enhancers. The utilization of these precursor compounds to augment NAD^+^ levels has the potential to yield numerous health advantages and medicinal applications [18, 38]. As opposed to other NAD^+^ precursors, NR is not associated with severe side effects or flushing and is thus currently regarded as a desirable precursor.

As a precursor of NAD^+^, NR has strong cell penetration and is not degradable by serum hydrolases [39]. NR is converted to NAD^+^ in two ways. First, NR is converted by NP to nicotinamide, which is converted by nicotinamide phosphoribosyltransferase to nicotinamide mononucleotide (NMN) in an ATP-dependent manner, which requires the participation of the phosphoribosyl diphosphate. NMN consumes ATP and is converted into NAD^+^ by NMNAT [40]. Second, NR is converted to NMN by NRK, and NMN is transformed to NAD^+^ by NMNAT [41].

In this study, we administered NR to mice with ICH, monitored NAD^+^ levels in the brain, and confirmed increased NAD^+^ levels following the administration of exogenous NR. Moreover, we found that NR protected against ICH and had a good safety profile.

NR inhibits the activation of NLRP3 induced by ICH. We quantified the expression level of NLRP3, Caspase-1, ASC, and COX-2 in the mouse tissue surrounding the right hematoma. NR substantially decreased the levels of NLRP3, Caspase-1, ASC, and COX-2, according to our findings. Moreover, we observed that NR significantly increased Nrf2 protein expression in the nucleus, but had no discernible effect on Nrf2 protein expression in the cytoplasm. In agreement with prior investigations, it has been determined that nuclear Nrf2 is the sole protein capable of stimulating the HO-1 gene and inhibiting cellular demise. Supplementation with NR promotes Nrf2 translocation from the cytoplasm to the nucleus, suggesting that the neuroprotective effects of NR against intracerebral hemorrhage-induced brain damage may be ascribed to enhanced nuclear Nrf2 activity. Furthermore, it was observed that exogenous NR substantially elevated the expression of HO-1, which indicates that NR may provide a safeguard against oxidative stress. The increase in ATP levels induced by NR in mice with ICH suggests that NR can sustain energy metabolism.

Several animal and human studies have confirmed that neuroinflammation plays a significant role in hemorrhagic brain injury and recovery [42]. The early stages of neuroinflammation involve microglia activation. The microglia cells were activated within 1 h of ICH, causing a series of inflammatory reactions, such as the release of inflammatory factors. Glial cell activation aggravates brain tissue injury in cerebral ischemia, and several studies have shown that inhibiting glial cell activation is beneficial [43, 44]. Based on previous studies, we examined glial cell activation after 24 h, and the results showed that inhibiting glial cell activation had a beneficial effect on brain injury following ICH. However, as glial cell activation can last for 3–4 weeks, further studies are needed to determine whether glial cell activation at different stages has beneficial or adverse effects on brain damage following ICH. In addition, we intend to conduct more comprehensive experiments due to insufficient sample size and possible bias during data collection and analysis. The primary objective of these investigations is to comprehensively examine the intricate molecular interactions, cellular reactions, and biochemical pathways by which Nicotinamide riboside (NR) imparts its protective effect in the context of hemorrhagic stroke. This will facilitate a more thorough investigation into the mechanisms by which NR exerts its effects.

**Fig. 11:**
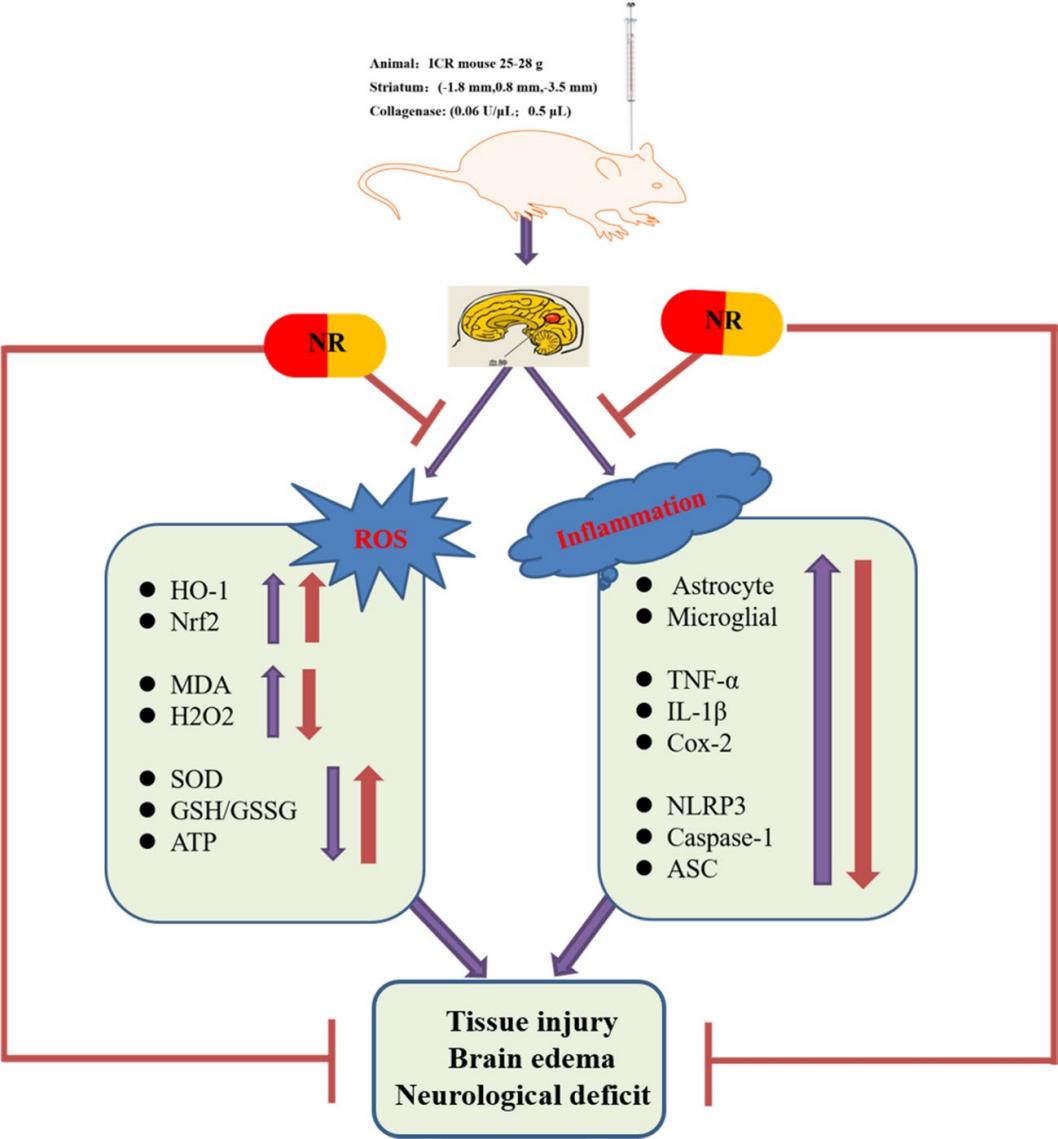
Therapeutic effect of NR on ICH

Summarily, this study demonstrated that NR has a protective effect against collagenase-induced hemorrhagic stroke. The protective effect of NR is mainly mediated by reduced inflammatory response and oxidative stress. NR is the precursor of NAD^+^, and NAD^+^ can be further converted to NADPH; NAD^+^ and NADPH may be effective drugs for ischemic and hemorrhagic stroke. Further studies are required to determine whether NAD^+^ and NADPH have a protective effect against hemorrhagic stroke to provide a scientific basis for developing NAD^+^ and NADPH as first-line drugs for the treatment of stroke. In a collagenase-induced model of hemorrhagic stroke in mice, NAD^+^ levels declined, neuroinflammation and oxidative stress increased, and neurological function was impaired. Exogenous NR exerted neuroprotective effects by inhibiting neuroinflammation and oxidative stress, thereby improving neurological function following ICH in mice (Fig. 11).

The diagram illustrates that NR reduces brain injury, cerebral edema, and neurological dysfunction by inhibiting oxidative stress, neuroinflammation, and release of proinflammatory factors.

## Abbreviations

NR: Nicotinamide Riboside
NAD^+^: Nicotinamide Adenine Dinucleotide
NADPH: Nicotinamide Adenine Dinucleotide Phosphate
ICH: Intracerebral hemorrhage
ROS: Reactive oxygen species
Nrf2: Nuclear respiration factor 2
GSH: Glutathione
SOD: Superoxide dismutase
MDA: Malondialdehyde
H_2_O_2_: Hydrogen peroxide
ATP: Adenosine triphosphate
NDS: Neurological dysfunction score

## References

[1] Li X, Zhu J, Dai L, Li M, Miao L, Liang J, Wang Y (2011) Trends in maternal mortality due to obstetric hemorrhage in urban and rural China, 1996-2005. J Perinat Med 39(1):35–41. 10.1515/jpm.2010.115 21138400 10.1515/jpm.2010.115

[2] Hostettler IC, Seiffge DJ, Werring DJ (2019) Intracerebral hemorrhage: an update on diagnosis and treatment. Expert Rev Neurother 19(7):679–694. 10.1080/14737175.2019.1623671 31188036 10.1080/14737175.2019.1623671

[3] Zeng J, Chen Y, Ding R, Feng L, Fu Z, Yang S, Deng X, Xie Z et al (2017) Isoliquiritigenin alleviates early brain injury after experimental intracerebral hemorrhage via suppressing ROS- and/or NF-kappaB-mediated NLRP3 inflammasome activation by promoting Nrf2 antioxidant pathway. J Neuroinflammation 14(1):119. 10.1186/s12974-017-0895-5 28610608 10.1186/s12974-017-0895-5 PMC5470182

[4] Babu R, Bagley JH, Di C, Friedman AH, Adamson C (2012) Thrombin and hemin as central factors in the mechanisms of intracerebral hemorrhage-induced secondary brain injury and as potential targets for intervention. Neurosurg Focus 32(4):E8. 10.3171/2012.1.FOCUS11366 22463118 10.3171/2012.1.FOCUS11366

[5] Wang G, Wang L, Sun XG, Tang J (2018) Haematoma scavenging in intracerebral haemorrhage: from mechanisms to the clinic. J Cell Mol Med 22(2):768–777. 10.1111/jcmm.13441 29278306 10.1111/jcmm.13441 PMC5783832

[6] Keep RF, Hua Y, Xi G (2012) Intracerebral haemorrhage: mechanisms of injury and therapeutic targets. Lancet Neurol 11(8):720–731. 10.1016/S1474-4422(12)70104-7 22698888 10.1016/S1474-4422(12)70104-7 PMC3884550

[7] Imai T, Iwata S, Hirayama T, Nagasawa H, Nakamura S, Shimazawa M, Hara H (2019) Intracellular Fe(2+) accumulation in endothelial cells and pericytes induces blood-brain barrier dysfunction in secondary brain injury after brain hemorrhage. Sci Rep 9(1):6228. 10.1038/s41598-019-42370-z 30996325 10.1038/s41598-019-42370-z PMC6470176

[8] Yang S, Chen Y, Deng X, Jiang W, Li B, Fu Z, Du M, Ding R (2013) Hemoglobin-induced nitric oxide synthase overexpression and nitric oxide production contribute to blood-brain barrier disruption in the rat. J Mol Neurosci 51(2):352–363. 10.1007/s12031-013-9990-y 23494638 10.1007/s12031-013-9990-y

[9] Katsu M, Niizuma K, Yoshioka H, Okami N, Sakata H, Chan PH (2010) Hemoglobin-induced oxidative stress contributes to matrix metalloproteinase activation and blood-brain barrier dysfunction in vivo. J Cereb Blood Flow Metab 30(12):1939–1950. 10.1038/jcbfm.2010.45 20354546 10.1038/jcbfm.2010.45 PMC2903654

[10] Xu F, Shen G, Su Z, He Z, Yuan L (2019) Glibenclamide ameliorates the disrupted blood-brain barrier in experimental intracerebral hemorrhage by inhibiting the activation of NLRP3 inflammasome. Brain Behav 9(4):e01254. 10.1002/brb3.1254 30859754 10.1002/brb3.1254 PMC6456786

[11] Liu CM, Shi BZ, Zhou JS (2014) Effects of thrombin on the secondary cerebral injury of perihematomal tissues of rats after intracerebral hemorrhage. Genet Mol Res 13(2):4617–4626. 10.4238/2014.June.18.4 25036511 10.4238/2014.June.18.4

[12] Ying W (2008) NAD+/NADH and NADP+/NADPH in cellular functions and cell death: regulation and biological consequences. Antioxid Redox Signal 10(2):179–206. 10.1089/ars.2007.1672 18020963 10.1089/ars.2007.1672

[13] Li M, Zhou ZP, Sun M, Cao L, Chen J, Qin YY, Gu JH, Han F et al (2016) Reduced Nicotinamide Adenine Dinucleotide Phosphate, a Pentose Phosphate Pathway Product, Might Be a Novel Drug Candidate for Ischemic Stroke. Stroke 47(1):187–195. 10.1161/STROKEAHA.115.009687 26564104 10.1161/STROKEAHA.115.009687

[14] Canto C, Menzies KJ, Auwerx J (2015) NAD(+) Metabolism and the Control of Energy Homeostasis: A Balancing Act between Mitochondria and the Nucleus. Cell Metab 22(1):31–53. 10.1016/j.cmet.2015.05.023 26118927 10.1016/j.cmet.2015.05.023 PMC4487780

[15] Huang Q, Sun M, Li M, Zhang D, Han F, Wu JC, Fukunaga K, Chen Z et al (2018) Combination of NAD(+) and NADPH Offers Greater Neuroprotection in Ischemic Stroke Models by Relieving Metabolic Stress. Mol Neurobiol 55(7):6063–6075. 10.1007/s12035-017-0809-7 29164394 10.1007/s12035-017-0809-7

[16] Zheng C, Han J, Xia W, Shi S, Liu J, Ying W (2012) NAD(+) administration decreases ischemic brain damage partially by blocking autophagy in a mouse model of brain ischemia. Neurosci Lett 512(2):67–71. 10.1016/j.neulet.2012.01.007 22260797 10.1016/j.neulet.2012.01.007

[17] Lautrup S, Sinclair DA, Mattson MP, Fang EF (2019) NAD(+) in Brain Aging and Neurodegenerative Disorders. Cell Metab 30(4):630–655. 10.1016/j.cmet.2019.09.001 31577933 10.1016/j.cmet.2019.09.001 PMC6787556

[18] Braidy N, Berg J, Clement J, Khorshidi F, Poljak A, Jayasena T, Grant R, Sachdev P (2019) Role of Nicotinamide Adenine Dinucleotide and Related Precursors as Therapeutic Targets for Age-Related Degenerative Diseases: Rationale, Biochemistry, Pharmacokinetics, and Outcomes. Antioxid Redox Signal 30(2):251–294. 10.1089/ars.2017.7269 29634344 10.1089/ars.2017.7269 PMC6277084

[19] Mehmel M, Jovanovic N, Spitz U (2020) Nicotinamide Riboside-The Current State of Research and Therapeutic Uses. Nutrients 12(6). 10.3390/nu12061616 10.3390/nu12061616 PMC7352172 32486488

[20] Han X, Bao X, Lou Q, Xie X, Zhang M, Zhou S, Guo H, Jiang G et al (2019) Nicotinamide riboside exerts protective effect against aging-induced NAFLD-like hepatic dysfunction in mice. PeerJ 7:e7568. 10.7717/peerj.7568 31523515 10.7717/peerj.7568 PMC6717504

[21] Lee HJ, Yang SJ (2019) Nicotinamide riboside regulates inflammation and mitochondrial markers in AML12 hepatocytes. Nutr Res Pract 13(1):3–10. 10.4162/nrp.2019.13.1.3 30788050 10.4162/nrp.2019.13.1.3 PMC6369115

[22] Vaur P, Brugg B, Mericskay M, Li Z, Schmidt MS, Vivien D, Orset C, Jacotot E et al (2017) Nicotinamide riboside, a form of vitamin B(3), protects against excitotoxicity-induced axonal degeneration. FASEB J 31(12):5440–5452. 10.1096/fj.201700221RR 28842432 10.1096/fj.201700221RR

[23] Hong G, Zheng D, Zhang L, Ni R, Wang G, Fan GC, Lu Z, Peng T (2018) Administration of nicotinamide riboside prevents oxidative stress and organ injury in sepsis. Free Radic Biol Med 123:125–137. 10.1016/j.freeradbiomed.2018.05.073 29803807 10.1016/j.freeradbiomed.2018.05.073 PMC6236680

[24] Li Q, Jiang X, Zhou Y, Gu Y, Ding Y, Luo J, Pang N, Sun Y et al (2023) Improving Mitochondrial Function in Skeletal Muscle Contributes to the Amelioration of Insulin Resistance by Nicotinamide Riboside. Int J Mol Sci 24(12). 10.3390/ijms241210015 10.3390/ijms241210015 PMC10297940 37373163

[25] Abulhasan YB, Teitelbaum J, Al-Ramadhani K, Morrison KT, Angle MR (2023) Functional Outcomes and Mortality in Patients With Intracerebral Hemorrhage After Intensive Medical and Surgical Support. Neurology 100(19):e1985–e1995. 10.1212/WNL.0000000000207132 36927881 10.1212/WNL.0000000000207132 PMC10186215

[26] Yang Y, Zhang Y, Wang Z, Wang S, Gao M, Xu R, Liang C, Zhang H (2016) Attenuation of Acute Phase Injury in Rat Intracranial Hemorrhage by Cerebrolysin that Inhibits Brain Edema and Inflammatory Response. Neurochem Res 41(4):748–757. 10.1007/s11064-015-1745-4 26498936 10.1007/s11064-015-1745-4

[27] Wang J, Dore S (2007) Inflammation after intracerebral hemorrhage. J Cereb Blood Flow Metab 27(5):894–908. 10.1038/sj.jcbfm.9600403 17033693 10.1038/sj.jcbfm.9600403

[28] Carbone F, Teixeira PC, Braunersreuther V, Mach F, Vuilleumier N, Montecucco F (2015) Pathophysiology and Treatments of Oxidative Injury in Ischemic Stroke: Focus on the Phagocytic NADPH Oxidase 2. Antioxid Redox Signal 23(5):460–489. 10.1089/ars.2013.5778 24635113 10.1089/ars.2013.5778 PMC4545676

[29] Qin YY, Li M, Feng X, Wang J, Cao L, Shen XK, Chen J, Sun M et al (2017) Combined NADPH and the NOX inhibitor apocynin provides greater anti-inflammatory and neuroprotective effects in a mouse model of stroke. Free Radic Biol Med 104:333–345. 10.1016/j.freeradbiomed.2017.01.034 28132925 10.1016/j.freeradbiomed.2017.01.034

[30] Aronowski J, Zhao X (2011) Molecular pathophysiology of cerebral hemorrhage: secondary brain injury. Stroke 42(6):1781–1786. 10.1161/STROKEAHA.110.596718 21527759 10.1161/STROKEAHA.110.596718 PMC3123894

[31] Zhu L, Sun S, Wu W, Zhang Y, Lin C, Ji L (2023) Xanthotoxol alleviates secondary brain injury after intracerebral hemorrhage by inhibiting microglia-mediated neuroinflammation and oxidative stress. Neurochirurgie 69(3):101426. 10.1016/j.neuchi.2023.101426 36921390 10.1016/j.neuchi.2023.101426

[32] Martinon F, Burns K, Tschopp J (2002) The inflammasome: a molecular platform triggering activation of inflammatory caspases and processing of proIL-beta. Mol Cell 10(2):417–426. 10.1016/s1097-2765(02)00599-3 12191486 10.1016/s1097-2765(02)00599-3

[33] Lin SJ, Ford E, Haigis M, Liszt G, Guarente L (2004) Calorie restriction extends yeast life span by lowering the level of NADH. Genes Dev 18(1):12–16. 10.1101/gad.1164804 14724176 10.1101/gad.1164804 PMC314267

[34] Zhang Q, Piston DW, Goodman RH (2002) Regulation of corepressor function by nuclear NADH. Science 295(5561):1895–1897. 10.1126/science.1069300 11847309 10.1126/science.1069300

[35] Shianna KV, Marchuk DA, Strand MK (2006) Genomic characterization of POS5, the Saccharomyces cerevisiae mitochondrial NADH kinase. Mitochondrion 6(2):94–101. 10.1016/j.mito.2006.02.003 16621727 10.1016/j.mito.2006.02.003

[36] Rajman L, Chwalek K, Sinclair DA (2018) Therapeutic Potential of NAD-Boosting Molecules: The In Vivo Evidence. Cell Metab 27(3):529–547. 10.1016/j.cmet.2018.02.011 29514064 10.1016/j.cmet.2018.02.011 PMC6342515

[37] Shabalin K, Nerinovski K, Yakimov A, Kulikova V, Svetlova M, Solovjeva L, Khodorkovskiy M, Gambaryan S et al (2018) NAD Metabolome Analysis in Human Cells Using (1)H NMR Spectroscopy. Int J Mol Sci 19(12). 10.3390/ijms19123906 10.3390/ijms19123906 PMC6321329 30563212

[38] Wei CC, Kong YY, Li GQ, Guan YF, Wang P, Miao CY (2017) Nicotinamide mononucleotide attenuates brain injury after intracerebral hemorrhage by activating Nrf2/HO-1 signaling pathway. Sci Rep 7(1):717. 10.1038/s41598-017-00851-z 28386082 10.1038/s41598-017-00851-z PMC5429727

[39] Schondorf DC, Ivanyuk D, Baden P, Sanchez-Martinez A, De Cicco S, Yu C, Giunta I, Schwarz LK et al (2018) The NAD+ Precursor Nicotinamide Riboside Rescues Mitochondrial Defects and Neuronal Loss in iPSC and Fly Models of Parkinson's Disease. Cell Rep 23(10):2976–2988. 10.1016/j.celrep.2018.05.009 29874584 10.1016/j.celrep.2018.05.009

[40] Katsyuba E, Auwerx J (2017) Modulating NAD(+) metabolism, from bench to bedside. EMBO J 36(18):2670–2683. 10.15252/embj.201797135 28784597 10.15252/embj.201797135 PMC5599801

[41] Canto C, Houtkooper RH, Pirinen E, Youn DY, Oosterveer MH, Cen Y, Fernandez-Marcos PJ, Yamamoto H et al (2012) The NAD(+) precursor nicotinamide riboside enhances oxidative metabolism and protects against high-fat diet-induced obesity. Cell Metab 15(6):838–847. 10.1016/j.cmet.2012.04.022 22682224 10.1016/j.cmet.2012.04.022 PMC3616313

[42] Hwang BY, Appelboom G, Ayer A, Kellner CP, Kotchetkov IS, Gigante PR, Haque R, Kellner M et al (2011) Advances in neuroprotective strategies: potential therapies for intracerebral hemorrhage. Cerebrovasc Dis 31(3):211–222. 10.1159/000321870 21178344 10.1159/000321870 PMC3721946

[43] Wang J (2010) Preclinical and clinical research on inflammation after intracerebral hemorrhage. Prog Neurobiol 92(4):463–477. 10.1016/j.pneurobio.2010.08.001 20713126 10.1016/j.pneurobio.2010.08.001 PMC2991407

[44] Deng S, Jin P, Liu S, He Y, Sherchan P, Zhang JH, Gong Y, Tang J (2023) Recruitment of regulatory T cells with rCCL17 promotes M2 microglia/macrophage polarization through TGFbeta/TGFbetaR/Smad2/3 pathway in a mouse model of intracerebral hemorrhage. Exp Neurol 367:114451. 10.1016/j.expneurol.2023.114451 37257716 10.1016/j.expneurol.2023.114451

